# Has Iran achieved the goal of reducing the prevalence of households faced with catastrophic health expenditure to 1%?: A national survey

**DOI:** 10.1002/hsr2.1199

**Published:** 2023-04-13

**Authors:** Azad Shokri, Amjad Mohamadi Bolbanabad, Satar Rezaei, Ghobad Moradi, Bakhtiar Piroozi

**Affiliations:** ^1^ Social Determinants of Health Research Center, Research Institute for Health Development University of Medical Sciences Kurdistan Sanandaj Iran; ^2^ Department of Public Health, School of Health, Research Center for Environmental Determinants of Health, Research Institute for Health Kermanshah University of Medical Sciences Kermanshah Iran

**Keywords:** catastrophic health expenditure, financial protection, health care expenditure, Iran

## Abstract

**Background and Aims:**

One of the goals of the Islamic Republic of Iran is to reduce the prevalence of catastrophic health expenditures among Iranian households to 1% by the end of the sixth 5‐year development plan (2016−2021). This study was conducted to evaluate the level of access to this goal in the final year of this program.

**Methods:**

A national cross‐sectional study was conducted on 2000 Iranian households in five provinces of Iran in 2021. Data were collected through interviews using the World Health Survey questionnaire. Data from households whose health care costs were more than 40% of their capacity to pay were included in the group of households with catastrophic health expanditure (CHE). Determinants of CHE were identified using univariate and multivariate regression analysis.

**Results:**

8.3% of households had experienced CHE. The variables of being a female head of household (odd ratio [OR] = 2.7), use of inpatient (OR = 1.82), dental (OR = 3.09), and rehabilitation services (OR = 6.12), families with disabled members (OR = 2.03) and low economic status of the households (OR = 10.73) were significantly associated with increased odds of facing CHE (*p* < 0.05).

**Conclusion:**

In the final year of the sixth 5‐year development plan, Iran has not yet achieved its goal of “reducing the percentage of households exposed to CHE to 1%.” Policymakers should pay attention to factors increasing the odds of facing CHE in designing interventions.

## 1 INTRODUCTION

According to the World Health Organization, one of the ultimate goals of health systems is to financially protect citizens against the catastrophic health expenditure.^1^ Households will face catastrophic health expanditure (CHE) when they spend more than 40% of their payment capacity out of pocket for receiving health services.^2^, ^3^ Health systems try to reduce the share of out‐of‐pocket payments (OOPs) when using health services by implementing mechanisms such as prepayments, subsidies, and free essential health services to increase financial protection. According to the World Health Organization, the CHE index in countries should be less than 1%.^4^, ^5^

Annually, a significant percentage of the world's population, often living in low and middle‐income countries, face catastrophic and impoverishing health expenditures due to the direct payment of health costs, or avoid receiving appropriate and timely health services.^6^, ^7^, ^8^ OOP of health expenditures is the most unfair way of financing health systems and brings about the highest risk for households to face CHE.^9^, ^10^ However, the main method of financing health systems in low and middle‐income countries is OOP.^11^

Universal health coverage and financial protection of citizens against catastrophic health expenditures is one of the obligations of many countries in the world including Islamic Republic of Iran.^12^ For this purpose, in Iran, in the law of the fourth (2005−2009), fifth (2011−2015), and sixth (2017−2021) 5‐year development plans, as one of the national strategic plans and upstream documents of the country, reducing the prevalence of Iranian households facing CHE to 1% has been set as one of the significant goals in these plans.^13^ In this regard, measures and policies such as increasing the percentage of population with basic health insurance, implementing rural family physician program and the health system transformation plan (as the latest reform in the country's health system whose details are given elsewhere) have been implemented so far.^12^, ^14^

However, at the end of the fourth and fifth development plans, this goal was not achieved and according to various national studies, there was a significant difference between the current situation and the predetermined goal (1%).^15^, ^16^, ^17^ According to the report of the research center of the Iranian parliament, the value of the CHE index for the country was reported 6% at the end of 2016 (final year of the fifth development plan).^18^ Therefore, the goal of reducing the percentage of households facing catastrophic health expenditures to less than 1% was again included in the agenda of the Iranian parliament and in the sixth development plan (Article 78).^13^ According to the studies conducted during the years of this program implementation, reaching this goal has not yet happened. Periodic measurement and regular monitoring of this index and its feedback to the policymakers of the health system is necessary to know the value of this index and implement the necessary reforms. We are now at the end of the sixth development plan and the present study aimed to evaluate the prevalence index of Iranian households facing CHE.

## 2 MATERIALS AND METHODS

### 2.1 Study design

This national cross‐sectional study was performed on Iranian households in 2021.

### 2.2 Data collection tools

World Health Survey Questionnaire developed by the World Health Organization was used to collect the data.^19^ This questionnaire has been translated in Iran and its validity and reliability have been confirmed in previous studies.^7^ This questionnaire was completed through an interview with the head of the household or an informed household member over 18 years of age. The questionnaire consists of three parts:

1. Background characteristics, demographic and socioeconomic status of the household.
2. Status of using outpatient health services (including visiting a doctor, psychologist, midwife and nurse as well as using medication and laboratory services), dental and rehabilitation services and hospitalization. The recall period for using outpatient, dental and rehabilitation services was 1 month (last 30 days) and the recall period for using inpatient services was 1 year (last 12 months).
3. Monthly household expenses by type of expense, including total household expenses, household food expenses and OOPs for health services. In this study, the recall period for total household expenses was 1 month (last 30 days).

### 2.3 Sampling and sample size

The sample size was estimated to be 385 households based on the following formula; taking into account the exposure of 50% of households to CHE (*p* = 0.5), 95% statistical significance, and 5% accuracy rate. Due to the fact that the study was conducted in 5 provinces, the total sample size was determined to be 2000 households (400 households from each province).

$$n=\frac{Z^{2}1-\frac{\alpha }{2}\times P(1-P)}{d^{2}}$$

Multi‐stage sampling was used to choose the samples. The country was divided into 5 geographical regions and one province was selected from each region. Five selected provinces included Tehran, Kurdistan, Golestan, Hormozgan, Kohgiluyeh and Boyer‐Ahmad. In the next stage, within each province, the capital city (county) of the province was selected. Then, within each county, the center and its villages were selected. Next, 10 centers were randomly selected from the comprehensive health centers in each city and 10 centers from rural health centers. Finally, from the list of households covered by each urban and rural center, 20 households were randomly selected and the questionnaire was completed by visiting the door of the household. The head of the clusters was considered as the first household.

### 2.4 Statistical analysis

#### 2.4.1 Economic status of households

The economic status of households was determined using the method proposed by O'Donnell et al.^20^ In this method, household assets including LCD TVs, separate refrigerators, washing machines, cell phones, dishwashers, microwave ovens, Internet access, private cars, private homes, and the number of rooms were assessed. Then, the asset index for each individual was calculated using principal component analysis and the study population was classified into 5 quantiles of 1 (the poorest), 2, 3, 4−5 (the richest).

In this study, the people of the society were divided into 3 categories in terms of socioeconomic status. Categories 1 and 2 were combined as the rich class (Q1), category 3 as the middle class of the society (Q2) and categories 4 and 5 were combined as the poor class (Q3).

In some studies, the asset index has been used to determine SES in the Iranian population.^21^, ^22^

#### 2.4.2 Catastrophic health care expenditure

In this study, the method provided by WHO was used to estimate the incidence of catastrophic health expenditures. Accordingly, if the amount of OOP for health expenses by family members is more than 40% of the households' capacity to pay, the household is in the group of households facing catastrophic health expenditures. Households' capacity to pay means effective income minus household living expenses. The method and details of CHE calculation are given in other studies.^2^

#### 2.4.3 Variables affecting catastrophic health care expenditure

Univariate logistic regression analysis was initially used to examine the role of determining variables (gender and age of the head of the household, basic and supplementary health insurance status, education and occupation of the head of the household, place of residence, use of inpatient, outpatient, dental and rehabilitation services, household size, household with a member more than 65 years old, having a disabled member, having a member under 5 years and economic status of the household) on the likelihood of experiencing CHE. In the adjusted model, first, by controlling the factors of age, sex, and education, the odds ratio of experiencing CHE was examined for each variable. Then, in the final model (stepwise), to obtain the desired model, after removing the independent variables that did not have much effect on the dependent variable, the odds of experiencing CHE for each variable in the presence of other variables were checked in the multivariate regression analysis model. It should be noted that the “use of outpatient services” was not included in the multivariate logistic model due to its collinearity with socioeconomic status. Also, it is worth mentioning that in Iran, health insurances are generally divided into two classes of basic and supplementary insurances. Basic insurances are governmental and supplementary insurances are private. Supplementary insurances are purchased to cover the patient's cost share and cover services outside the service package of basic insurances. In Iran, some services such as dental and rehabilitation services are not covered by basic insurance. All tests were performed in STAT 12.0 software (Stata Corporation) and the significance level was set at *p* < 0.05.

## 3 RESULTS

A total of 1926 households from five provinces of the country participated in this study, of which 90% (1739 people) were male heads of households and most had primary or middle school education (72%), were employed (75%) and only 5.8% (112 people) had supplementary insurance. In this study, the rate of exposure to CHE was reported to be 8.3%, which was significantly higher among users of rehabilitation services (35%), households with a socioeconomic status lower than average (27%), households with disabled members (22%), users of dental services (20%), female head of the household (17%) and being a housewife (17%) (*p* < 0.05). It should be noted that there was no difference in exposure to CHE between the studied provinces, educational groups, basic insurance coverage status, urban or rural status of the households, and household size (*p* > 0.05) (Table 1).

**Table 1:** Demographic characteristics of households by catastrophic health expenditure exposure status

	Variables		Faced with catastrophic health expenditures		
		Number (%)	No *n* (%)	Yes *n* (%)	*p* Value
Sex of household head					***<0.001***
	Male	1739 (90.3)	1611 (92.6)	128 (7.4)	
	Female	187 (9.7)	155 (82.9)	32 (17.1)	
Age of household head					***0.001***
	<40	780 (40.5)	707 (90.6)	73 (9.4)	
	40−50	713 (37.0)	643 (90.2)	70 (9.8)	
	50<	433 (22.5)	416 (96.7)	17 (8.3)	
Insurance coverage					0.563
	Yes	1874 (97.3)	1718 (91.7)	156 (8.3)	
	No	52 (2.7)	48 (92.3)	4 (7.7)	
Supplementary insurance coverage					***0.001***
	Yes	112 (5.8)	111 (99.1)	1 (0.9)	
	No	1813 (94.2)	1654 (91.2)	159 (8.8)	
Education status of the household head					0.224
	Illiterate	161 (8.4)	143 (88.8)	18 (11.2)	
	Primary school	801 (41.6)	726 (90.6)	75 (9.4)	
	Middle school	579 (30.1)	536 (92.6)	43 (7.4)	
	High school	226 (11.7)	212 (93.8)	14 (6.2)	
	University	159 (8.3)	149 (93.7)	10 (6.3)	
Job of household head					***0.008***
	Has job	1444 (75.0)	1337 (92.6)	107 (7.4)	
	Retired	93 (4.8)	88 (94.6)	5 (5.4)	
	Housewife	23 (1.2)	19 (82.6)	4 (17.4)	
	Unemployed	366 (19.0)	322 (88.0)	44 (12.0)	
Place of residence					0.171
	Urban	1002 (52.0)	925 (92.3)	77 (7.7)	
	Rural	924 (48.0)	841 (91.0)	83 (9.0)	
Utilization of Inpatient Services					***0.007***
	No	1752 (91.0)	1616 (92.2)	136 (7.8)	
	Yes	174 (9.0)	150 (86.2)	24 (13.8)	
Utilization of outpatient services					***<0.001***
	No	913 (47.4)	908 (99.5)	5 (0.5)	
	Yes	654 (52.6)	857 (84.7)	155 (15.3)	
Utilization of dental services					***<0.001***
	No	1765 (91.6)	1604 (93.1)	119 (6.9)	
	Yes	161 (8.4)	162 (79.8)	41 (20.2)	
Utilization of rehabilitation services					***<0.001***
	No	1832 (95.1)	1705 (93.1)	127 (6.9)	
	Yes	94 (4.9)	61 (64.9)	33 (35.1)	
Household size					0.064
	4>	1076 (55.9)	977 (90.8)	99 (9.2)	
	4<	203 (10.5)	789 (92.8)	61 (7.2)	
	No	1860 (96.6)	1727 (92.8)	133 (7.2)	
	Yes	66 (3.4)	39 (59.1)	27 (40.9)	
Household having member over 65 years old					***<0.001***
	No	1483 (77.0)	1357 (91.5)	126 (8.5)	
	Yes	443 (23.0)	409 (92.3)	34 (7.7)	
Household having member(s) with disabilities					***<0.001***
	No	1799 (93.4)	1668 (92.7)	131 (7.3)	
	Yes	127 (6.6)	98 (77.2)	29 (22.8)	
Household having member(s) under 5 years old					0.231
	No	1425 (74.0)	1311 (92.0)	114 (8.0)	
	Yes	501 (26.0)	455 (90.8)	46 (9.2)	
Socioeconomic status					***<0.001***
	Q1 (the poorest)	664 (34.5)	569 (85.7)	95 (14.3)	
	Q2	441 (22.9)	391 (88.7)	50 (11.3)	
	Q3 (the richest)	821 (42.6)	806 (98.2)	15 (1.8)	
Province					0.921
	Tehran	383 (19.9)	355 (92.7)	28 (7.3)	
	Golestan	392 (20.4)	360 (91.8)	32 (8.2)	
	BandarAbas	382 (19.8)	350 (91.6)	32 (8.4)	
	BoyehAhmad	381 (19.8)	346 (90.8)	35 (9.2)	
	Kurdistan	388 (20.1)	355 (91.5)	33 (8.5)	
Total		**1926**	**1766 (91.7)**	**160 (8.3)**	

*Note*: Bold values indicate statistically significant *p* < 0.05.

Abbreviations: ANOVA, analysis of variance.

^a^ Obtained from ANOVA.

Average household total monthly expense was 30.07 ± 10.15 million Iranian Rial (122.8 US$) and average household total monthly health expenditure was 1.970 ± 1.180 million Iranian Rial (7.89 US$).

Table 2 shows the determinants of CHE exposure in the crude and adjusted models. In the crude model, the majority of variables were among the determinants of experiencing CHE (*p* < 0.05).

**Table 2:** Multivariate logistic regression results for determinants of exposure to CHE

		Model 1		Model 2		Model 3		Model 4	
		OR (95% CI)	*p* Value	OR (95% CI)	*p* Value	OR (95% CI)	*p* Value	OR (95% CI)	*p* Value
Sex of household head									
	Male	1.00				1.00		1.00	
	Female	2.59 (1.71−3.96)	***<0.001***	‐		2.87 (1.76−4.68)	***<0.001***	2.75 (2.75−1.64)	***<0.001***
Age of household head									
	<40	1.00							
	40−50	1.05 (0.74−1.49)	0.763	‐					
	50<	0.39 (0.23−0.68)	***<0.001***	‐					
Insurance coverage									
	Yes	1.00		1.00					
	No	0.87 (0.32−2.57)	0.871	0.92 (0.32−2.60)	0.918				
Supplementary insurance coverage									
	Yes	1.00		1.00		1.00			
	No	5.25 (1.28−21.45)	***0.021***	4.35 (1.05−18.08)	***0.043***	0.32 (0.48−2.68)	0.317		
Education status of the household head									
	Illiterate	1.00							
	Primary school	0.82 (0.47−1.42)	0.477	‐					
	Middle school	0.64 (0.35−1.14)	0.128	‐					
	High school	0.52 (0.25−1.09)	0.083	‐					
	University	0.53 (0.23−1.19)	0.126	‐					
Job of household head									
	Has job	1.00		1.00					
	Retired	0.71 (0.28−1.79)	0.467	0.90 (0.34−2.35)	0.825				
	Housewife	2.63 (0.88−7.88	0.084	1.65 (0.51−5.34)	0.404				
	Unemployed	1.71 (1.18−2.47)	***0.005***	1.97 (1.34−2.88)	***0.001***				
Place of residence									
	Urban	1.00		1.00					
	Rural	1.18 (0.86−1.64)	0.303	1.25 (0.89−1.74)	0.19				
Utilization of Inpatient Services									
	No	1.00		1.00		1.00		1.00	
	Yes	1.99 (1.26−3.15)	***0.003***	1.68 (1.05−2.69)	***0.032***	2.144 (1.28−3.59)	***0.004***	1.82 (1.05−3.15)	***0.032***
Utilization of outpatient services									
	No	1.00		1.00					
	Yes	12.73 (7.02−23.10)	***<0.001***	12.73 (7.02−23.10)	***<0.001***	‐		‐	
Utilization of dental services									
	No	1.00		1.00		1.00		1.00	
	Yes	3.41 (2.31−5.04)	***<0.001***	3.17 (2.13−4.71)	***<0.001***	3.28 (2.14−5.03)	***<0.001***	3.09 (1.99−4.80)	***<0.001***
Utilization of rehabilitation services									
	No	1.00		1.00		1.00		1.00	
	Yes	7.26 (4.58−11.51)	***<0.001***	7.01 (4.39−11.18)	***<0.001***	5.86 (3.30−10.41)	***<0.001***	6.12 (3.38−11.11)	***<0.001***
Household size									
	4>	1.00		1.00		1.00			
	4<	0.76 (0.55−1.60)	0.11	0.87 (0.62−1.23)	0.451	0.86 (0.59−1.23)	0.400		
Household having member over 65 years old									
	No	1.00		1.00					
	Yes	0.89 (0.60−1.32)	0.58	0.83 (0.56−1.24)	0.372				
Household having member(s) with disabilities									
	No	1.00		1.00		1.00		1.00	
	Yes	3.76 (2.40−5.91)	***<0.001***	3.75 (1.37−5.95)	***<0.001***	3.38 (1.38−4.10)	***0.002***	2.30 (1.32−4.02)	***0.003***
Household having member(s) under 5 years old									
	No	1.00		1.00					
	Yes	1.16 (0.81−1.66)	0.41	1.11 (0.76−1.58)	0.61				
Socioeconomic status									
	Q3 (the richest)	1.00		1.00		1.00		1.00	
	Q2	6.87 (3.81−12.39)	***<0.001***	6.71 (3.69−12.21)	***<0.001***	7.06 (3.85−12.95)	***<0.001***	7.12 (3.82−13.25)	***<0.001***
	Q1 (the poorest)	8.97 (5.15−15.63)	***<0.001***	10.05 (5.72−17.64)	***<0.001***	10.18 (5.73−18.10)	***<0.001***	10.73 (6.00−19.19)	***<0.001***
Province									
	Tehran	1.00							
	Golestan	1.13 (0.66−1.91)	0.657	1.04 (0.61−1.77)	0.884				
	BandarAbas	1.15 (0.68−1.96)	0.584	1.07 (0.63−1.83)	0.800				
	BoyehAhmad	1.28 (0.76−2.15)	0.347	1.14 (0.67−1.93)	0.624				
	Kurdistan	1.18 (0.69−1.99)	0.539	1.09 (0.64−1.85)	0.762				

*Note*: Model 1: Crude model; Model 2: Adjusted model by age, sex and education level; Model 3: Adjusted model by removing all variables with *p* > 0.2; Model 4: The stepwise regression method by adding or removing predictor variables to obtain the most parsimonious model. Bold values indicate statistically significant *p* < 0.05.

For instance, the odds for female heads facing CHE were 2.6 times higher than male heads (*p* < 0.001, 95% CI: 1.71−3.96), for households without supplementary insurance were 5.3 times (*p* < 0.001, 95% CI: 1.71−3.96), for users of outpatient services were 12.7 times (*p* < 0.001, 95% CI: 7.02−23.10), for users of rehabilitation services were 7.3 times (*p* < 0.001, 95% CI: 4.58−11.51), for users of inpatient services were 1.99 times (*p* = 0.003, 95% CI: 1.26−3.15), for households with lower socioeconomic status were 9 times (*p* < 0.001, 95% CI: 5.15−15.63), and for households with a disabled member were 3.8 times (*p* < 0.001, 95% CI: 5.33−15.14).

In the first adjusted model (by controlling factors of age, sex, and education), the odds of encountering CHE in a household without supplementary insurance (OR = 4.35), in users of outpatient (OR = 12.73), hospitalization (OR = 1.68), rehabilitation (OR = 7.01), and dental services (OR = 3.17), in households with a disabled member (OR = 3.75), and in households with a low socioeconomic status (OR = 10.18) were reported to be significantly higher (*p* < 0.05).

In the final models, to obtain the desired model by removing the independent variable that had little effect on the dependent variable, gender of head of the household (OR = 2.75), use of inpatient (OR = 1.82), dental (OR = 3.09), and rehabilitation (OR = 6.12) services, households with disabled members (OR = 2.03), and socioeconomic status of households (OR = 10.73 and 7.12) as final variables were significantly associated with household exposure to CHE (*p* < 0.05).

## 4 DISCUSSION

Universal health coverage means that everyone has access to health services they need without facing financial barriers.^23^ According to the World Health Organization, the CHE value should be less than 1%.^4^ Achieving universal health coverage is one of the commitments of the Islamic Republic of Iran. In this regard, reducing the CHE index to 1% has been stated as a goal in the fourth, fifth and sixth 5‐year development plans of the country.^13^

Based on the findings of this study, in the last year of the sixth 5‐year development plan (2017−2021), like the two previous development plans, Iran not only did not achieve the goal of reducing the percentage of households exposed to CHE to 1%, but also, compared to the findings of previous studies, this gap seems to have become deeper.^15^, ^17^ The coincidence of the last 2 years of this program with the start of the Covid‐19 pandemic in the world and in Iran may have been one of the factors affecting it. Covid‐19 pandemic has affected the CHE index in various ways, such as increasing health costs, increasing unemployment, weakening the household's economic status, and reducing the household's ability to pay.^24^, ^25^, ^26^ In addition, in recent years, Iran has had a growing inflation, which can also be a reason for that.^27^, ^28^, ^29^

According to the findings of another systematic and meta‐analysis study in Iran (2017), the prevalence of households faced with CHE was reported to be 3.9% (95% CI, 3.26−11.07).^30^ Also, in the latest systematic and meta‐analysis study in Iran (2018), in total, the percentage of households exposed to CHE was 7.5% (95% CI, 6.2−9.1) and for inpatient services was 35.9% (95% CI, 23.5−54.3).^15^ Also, according to the report of the research center of the Iranian parliament, the value of the CHE index for the country at the end of 2016 (end of the fifth development plan) was reported 6%.^18^ A comparison of the findings of our study with the findings of the preceding studies shows that the status of the financial protection index against CHE in Iran has become even worse.^15^, ^17^, ^30^ Designing appropriate and targeted interventions to move towards achieving universal health coverage, increasing the government's share of health costs, reducing the share of OOPs for health services, revising list of services covered by basic health insurance, dedicating targeted health subsidies for poor households, and more financial protection should be on the agenda of policymakers of Iran's health system.

According to our study, in final models, variables of being female head of household, use of inpatient, outpatient, dental, and rehabilitation services, families with disabled members and households with low socioeconomic status, were significantly associated with increased odds of facing CHE.

In most similar studies conducted in Iran, these variables have been reported as variables affecting the CHE.^7^, ^31^, ^32^ In Iran, despite the fact that a high percentage of population is covered by basic health insurance (nearly 90%), service coverage and depth of coverage of health costs are not in a good situation and these insurance companies have not been able to provide adequate financial protection of people against health costs and a significant part of the costs is paid out of patients' pockets.^33^ In Iran, dental and rehabilitation services and equipment are not covered by basic health insurance and most of these services are also provided by the private sector that it leads to an increase in household health costs and subsequently increases the risk of CHE. In most studies, the use of rehabilitation and dental services have been reported as determinants of exposure to CHE.^7^ The results of a systematic and meta‐analysis study in Iran (2021) showed that health insurance companies do not provide effective financial protection against CHE.^33^ Based on the results of the preceding study, the expansion of prepayment mechanisms, integration of insurance funds and designing insurance service packages to cover chronic diseases have been proposed as appropriate solutions for financial protection against CHE.^33^ According to findings of various studies in Iran, the variables of using inpatient, outpatient, and dental services, low education, rural life and low economic status of the household, being a female head of the household, not having insurance, having a child under 5 years, having an elderly person and a person with a disability in the household are the factors that determine CHE in Iran.^30^, ^34^

According to our study, having supplementary insurance did not lead to greater financial protection and nor reduced the odds of facing CHE. This finding contradicts the findings of many previous studies.^6^, ^10^, ^16^ This lack of effectiveness of supplementary insurance to protect people from catastrophic health costs can be caused by the effect of household expenses spent on using means to prevent the spread of Covid‐19 and the weakening of the economic status of households due to Covid‐19 pandemic and the subsequent reduction in households' capacity to pay that makes them vulnerable to health costs.

### 4.1 Strengths of the study

This study was conducted at national level and is the first study at this level to evaluate the goal related to the financial protection of citizens against CHE in the final year of the sixth 5‐year development plan of the Islamic Republic of Iran.

### 4.2 Limitations of the study

In this study, data were collected based on self‐declaration of individuals, so the recall bias may have occurred about the household expenses, health care costs and the type of health services consumed, although we tried to reduce this type of bias by shortening the recall period.

## 5 CONCLUSION

The findings of this study indicated that in the last year of the sixth 5‐year development plan, Iran has not achieved the goal of “reducing the percentage of households exposed to CHE to 1%.” Policymakers should pay attention to factors increasing the chance of facing CHE in designing interventions and this can help the goal of financial protection against health costs. Increasing the government's share of total health costs, expanding appropriate prepayment mechanisms, revising service packages under insurance coverage and covering services such as dental and rehabilitation services, increasing the depth of coverage of health costs by insurance companies, payment exemptions for poor households and households with a disabled person can boost the financial protection of households against CHE.

## AUTHOR CONTRIBUTIONS

**Azad Shokri**: Conceptualization; data curation; formal analysis; methodology; writing—original draft. **Amjad Mohamadi Bolbanabad**: Data curation; supervision; writing—original draft. **Satar Rezaei**: formal analysis; methodology; writing—original draft. **Bakhtiar Piroozi**: Conceptualization; formal analysis; methodology; supervision; writing—original draft.

## CONFLICT OF INTEREST STATEMENT

The authors declare no conflict of interest.

## ETHICS STATEMENT

This study was approved by ethics committee of Kurdistan University of Medical Sciences with the code IR.MUK.REC.1399.076. Participation in the study was voluntary and written consent was obtained from the participants.

## TRANSPARENCY STATEMENT

The lead author Bakhtiar Piroozi affirms that this manuscript is an honest, accurate, and transparent account of the study being reported; that no important aspects of the study have been omitted; and that any discrepancies from the study as planned (and, if relevant, registered) have been explained.

## Data Availability

The data that support the findings of this study are available from the corresponding author (Bakhtiar Piroozi) upon reasonable request.
